# A spatial analysis of the epidemiology of HIV-infected students in Zhejiang province, China

**DOI:** 10.1186/s12879-021-06033-7

**Published:** 2021-05-07

**Authors:** Wanjun Chen, Jiezhe Yang, Jun Jiang, Lin He, Yun Xu, Jinlei Zheng, Jianmin Jiang, Xiaohong Pan

**Affiliations:** Department of AIDS and STD Prevention and Control, Zhejiang Provincial Center for Disease Control and Prevention, Hangzhou, 310051 China

**Keywords:** Acquired immunodeficiency syndrome, HIV-infected students, Men who have sex with men

## Abstract

**Background:**

The upsurge in HIV infections among students is a matter of particular concern. However, few studies have explored the epidemiological characteristics including the risky sexual networking of HIV-infected students in Zhejiang province, China.

**Methods:**

Using the provincial surveillance data of HIV-infected students, we conducted a retrospective epidemiology study to describe the epidemiological characteristics of 628 newly diagnosed cases from 2011 to 2016 and detailed information of 124 cases from 2015 to 2016. Spatial analyses were conducted using ArcGIS software, and statistical analyses were performed using SPSS software.

**Results:**

A total of 628 cases of HIV/AIDS were diagnosed among students in Zhejiang Province, China between 2011 and 2016. The cases showed an overall increasing trend over time, while the proportions of students with HIV disease status, cases diagnosed by HIV voluntary counseling and testing (VCT), and cases of homosexual transmission remained stable over time. Significant spatial heterogeneity in the cases was seen at the county level. Detailed data on 124 HIV-positive individuals collected from the local Center for Disease Control and Prevention (CDC) from 2015 and 2016, showed that the majority of them (85.5%,) engaged in homosexual behavior, and 93.4% had sex with casual partners. These partners included not only social members, but also other students. Online dating applications represented the most common means of seeking and communicating with homosexual partners. The level of awareness regarding the risk of HIV infection, and the amount coverage of face-to-face education towards students were both low.

**Conclusions:**

HIV infections among students were characterized by increasing trend and spatial clustering in Zhejiang Province between 2011 and 2016, with homosexual sexual activity being the main mode of infection. Interventions are urgently required to prevent HIV infection in this population by increasing awareness of the disease. HIV testing programs and information regarding disease prevention specifically through online dating applications are needed.

## Background

Sexual contact is the primary mode of human immunodeficiency virus (HIV) in China: sexual transmission accounted for 95.1% of the total cases in 2017 [1, 2]. Heterosexual transmission accounted for 69.6% of these cases, with homosexual transmission accounted for 25.5% [2]. The upsurge in HIV cases among students is a matter of particular concern [3]. The number of newly diagnosed cases of HIV infection among college students in 2017 (3077 cases) was more than tenfold greater than that in 2006 (242 cases), with an annual increase ranging from 30 to 50% over that period [2–4].

The increase in HIV cases epidemic among students has been identified as one of the major challenges in the overall HIV prevention strategy for China [3]. Several factors increase the risk of HIV infection among students, including inadequate sex education at school, earlier onset of sexual activity, and low awareness of methods of self-protection [5, 6]. Another critical factor is the use of mobile phones and social media among students; these platforms have increased the number of people with whom students can interact socially [7]. Individuals aged 18 to 25 years account for 50% of the users of the largest gay dating application in China (Blued) [8]. Studies have also shown that young MSM are more likely to seek partners online and engaged in high-risk sexual behaviors with partners met via Internet [9, 10]. Previous studies of students have mainly investigated attitudes and knowledge regarding HIV and sexual behavior, or compared disease prevalence between men who have sex with men (MSM) and those who do not [11–14]. Few analyses of “risky sexual networking” among HIV-infected students have been performed [4, 15, 16]. Moreover, few studies have attempted to assess the risk of infection, or describe the epidemiological characteristics of HIV-infected students in detail.

Spatial analysis has been commonly applied in HIV research to assess the long-term trends and geographic distribution patterns. Qin performed a spatial analysis of HIV-infected MSM in China from 2006 to 2015 [17]. Zulu described the temporal and spatial distribution of HIV prevalence in Malawi from 1994 to 2010 [18]. Spatial patterns of HIV infection among students are important to understand for planning, future public health programs and targeted interventions.

This study was performed to better understand epidemiological trends of HIV infection among students in Zhejiang Province, China between 2011 and 2016.

## Materials and methods

### Data collection

Annual data on HIV-infected students from 2011 to 2016 were obtained from the National Data Information System for Comprehensive HIV/AIDS Control database for Zhejiang Province. HIV-infected students in this study were defined as HIV-positive individuals who were students and diagnosed by physicians at the medical institutions in Zhejiang Province based on national standards [19–21]. Blood samples were initially screened for HIV by enzyme-linked immunosorbent assay (ELISA). Positive results were confirmed by western blot. Data on the sociodemographic characteristics, disease status, routes of transmission, and risky behaviors were collected by the staff of the local Center for Disease Control and Prevention (CDC) using standardized forms and face-to-face interviews conducted in a private room. All data used in this study were anonymized to protect the privacy of participants.

We also collected detailed epidemiological data on students diagnosed with HIV in the period 2015–2016 from the local CDC, including on sexual behavior (homosexual or heterosexual sex with different partners), history of HIV testing, perspective of HIV-infected risk and knowledge about precautions against HIV infection. With regard to partners, a commercial partner defined as someone the student bought sex from or sold sex to; a casual partner defined as someone the student knew and had sex with occasionally; a regular partner defined as someone the student had sex with on an ongoing basis. With regard to homosexual behaviors, the information about condom use, and use of online dating applications were collected.

### Data analyses

The annual number of cases of HIV infection among students was plotted against transmission route. The proportions according to HIV disease status, and source of infection were calculated and plotted to reveal the variation in the number of cases.

Descriptive analyses were conducted to elucidate sexual risk behaviors and interventions status for the detailed characteristics of HIV-infected students. All descriptive statistical analyses were performed using SPSS software (version 18.0; IBM Corp., Armonk, NY, USA). The significance level was defined as *p* < 0.05 at 95% confidence intervals.

The spatial distribution of HIV infections among students was mapped on an annual basis using ArcGIS software (version 10.2; ESRI Inc., Redlands, CA, USA), from 2011 to 2016 at the county level of Zhejiang Province by the study group. A two-phases spatiotemporal analysis was performed for each year. First, a global test (Moran’s *I* index) was used to assess spatial autocorrelations in annual trends of cases during the study period. A negative value of Moran’s *I* indicates a dispersed distribution pattern, while a positive value indicates a clustered distribution. A value around zero indicates a spatially random distribution. Second, local indicators of spatial association were examined to explore the spatiotemporal clustering of HIV/AIDS cases. Significant hotspots (High-High), coldspots (Low-Low), and outliers (High-Low and Low-High) by calculating local Moran’s *I* index between a given location and the average of neighboring values in the surrounding locations. We performed the local test for each year at the county level during the study period. These analyses were both performed using ArcGIS software (version 10.2; ESRI Inc., Redlands, CA, USA).

## Results

### Distribution patterns of HIV infections among students, 2011–2016

**Fig. 1:**
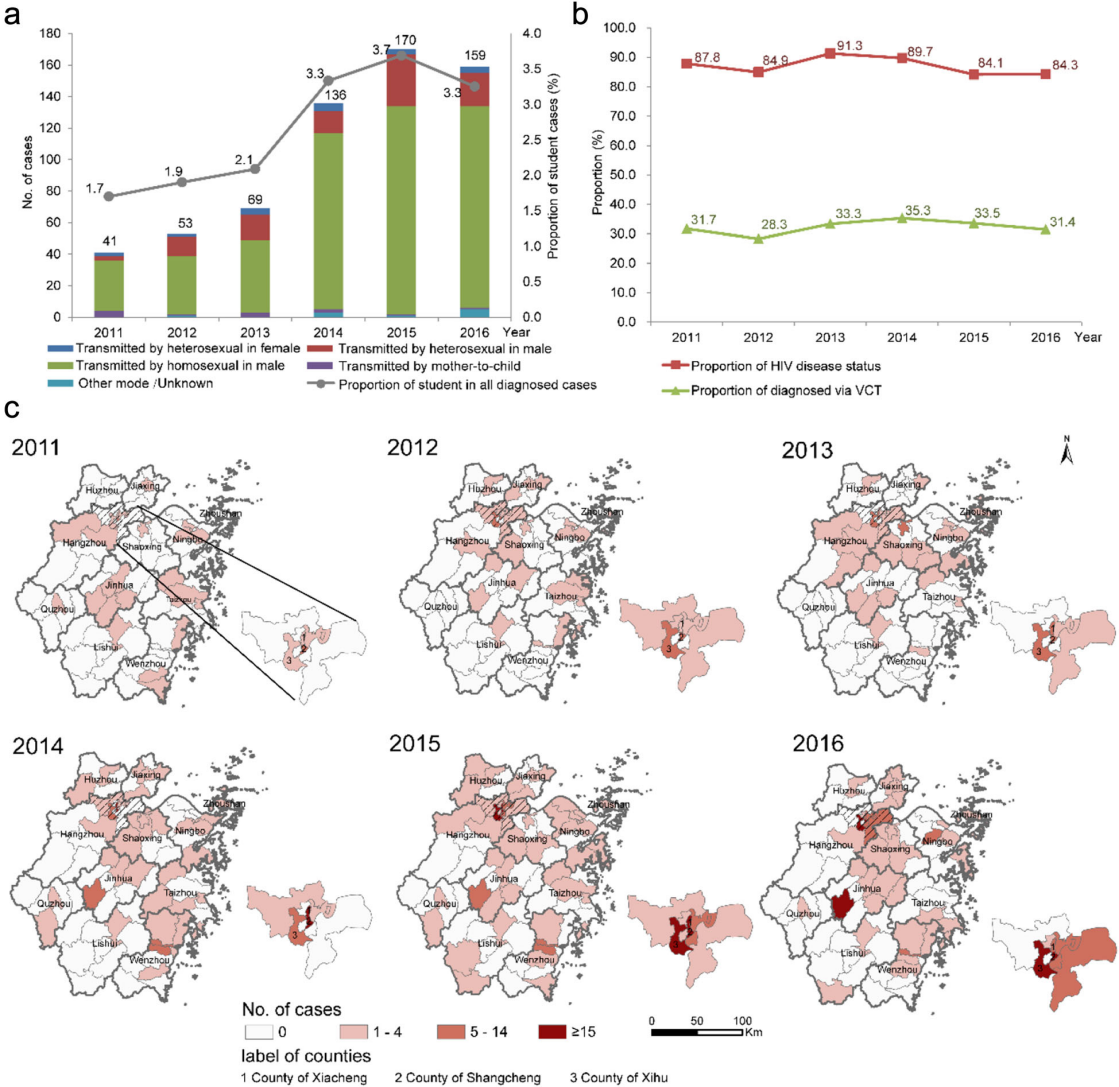
The distributional patterns of HIV/AIDS among students, 2011–2016. **a** The temporal trend and the transmission route; **b** The social-demographical characteristics; **c** The annually spatial distribution of cases. The map depicted in the figure is our own

There were 628 cases of HIV infections among students in Zhejiang Province, China in the period 2011–2016, with 41, 53, 69, 136, 170, and 159 cases in the respective years. The proportion of students with HIV/AIDS among all cases diagnosed in Zhejiang increased from 1.7% in 2011 to 3.3% in 2016 over the study period (Cochran-Armitage trend test, *p* < 0.01). The proportion of individuals infected through homosexual behavior was 77.5%, and that of 15.8% through heterosexual behaviors in male, being both no significant differences in 2011 to 2016 (Cochran-Armitage trend test, *p* > 0.05, Fig. 1a). The proportions of individuals with HIV disease status, and a diagnosis of HIV obtained via voluntary counseling and testing (VCT) also showed no significant changes over time (Cochran-Armitage trend test, *p* > 0.05, Fig. 1b).

### Detailed information on HIV-infected students collected from the local CDC

A total of 124 students diagnosed from 2015 to 2016 (37.7% of the newly diagnosed cases) participated in a supplementary study. Compared to the HIV-infected students in the same period, there were no statistically significant differences in age, gender, marital status, or education level (chi-square test, *p* > 0.05). Of the 124 students, 122 (98.4%) were male, and only 2 (1.6%) were female. In total, 57 (46.0%) did not believe that they could be infected with HIV, and 54 (43.6%) students diagnosed with HIV were not familiar with precautionary measures against HIV infection (Table 1).

**Table 1:** The characteristics of HIV-infected students with detailed investigation

Variables	Cases(n)	Percentage(%)
**Age (Mean, SD)**	20.9 (2.4)	
**Marital status**		
Single	123	99.2
Other	1	0.8
**Education**		
Junior high	2	1.6
Denior high	11	8.9
College or above	111	89.5
**Ethnicity**		
Han	121	97.6
Other	3	2.4
**Diagnosed source**		
VCT	49	39.5
Clinic	56	45.2
Other	19	15.3
**The perspective of HIV-infected risk**		
Impossible	57	46.0
Possible	67	54.0
**Ever tested for HIV previously**		
Yes	53	42.7
No	71	57.3
**Being familiar with the precautions against HIV infection**		
Yes	70	56.5
No	54	43.5
**Type of sexual behavior**		
Male homosexual behavior	98	79.0
Male homosexual behavior + male heterosexual behavior	8	6.5
Male heterosexual behavior	16	12.9
Female heterosexual behavior	2	1.6

The study population included 106 (85.5%) male students who reported engaging in homosexual behavior, among whom 98 reported having engaged only in homosexual intercourse. With regard to homosexual intercourse, 99 (93.4%) had had sex with casual partners (along with commercial partners in 3 cases, along with regular partners in 33 cases), 7 students had sex only with regular partners. Only 9 cases used condoms consistently during homosexual behaviors with partners in the preceding 1 year before HIV diagnosed. In total, 18.3% (data were missing for 2 students) had other students as partners, while 37.5% had social members as partners, and 44.2% had both types of partners. Among these respondents, 80 (81.6%) students reported seeking and communicating with partners using geosocial networking applications targeting MSM such as Blued and Jack’d, while 54 (54.0%) used social media messaging platforms, such as QQ, WeChat and Momo, 18 (18.4%) by venues prepared for MSM, 25 (25.5%) communicated by telephone, and 7 (7.0%) communicated via other modalities.

### Spatial analyses of HIV infection among students, 2011–2016

**Fig. 2:**
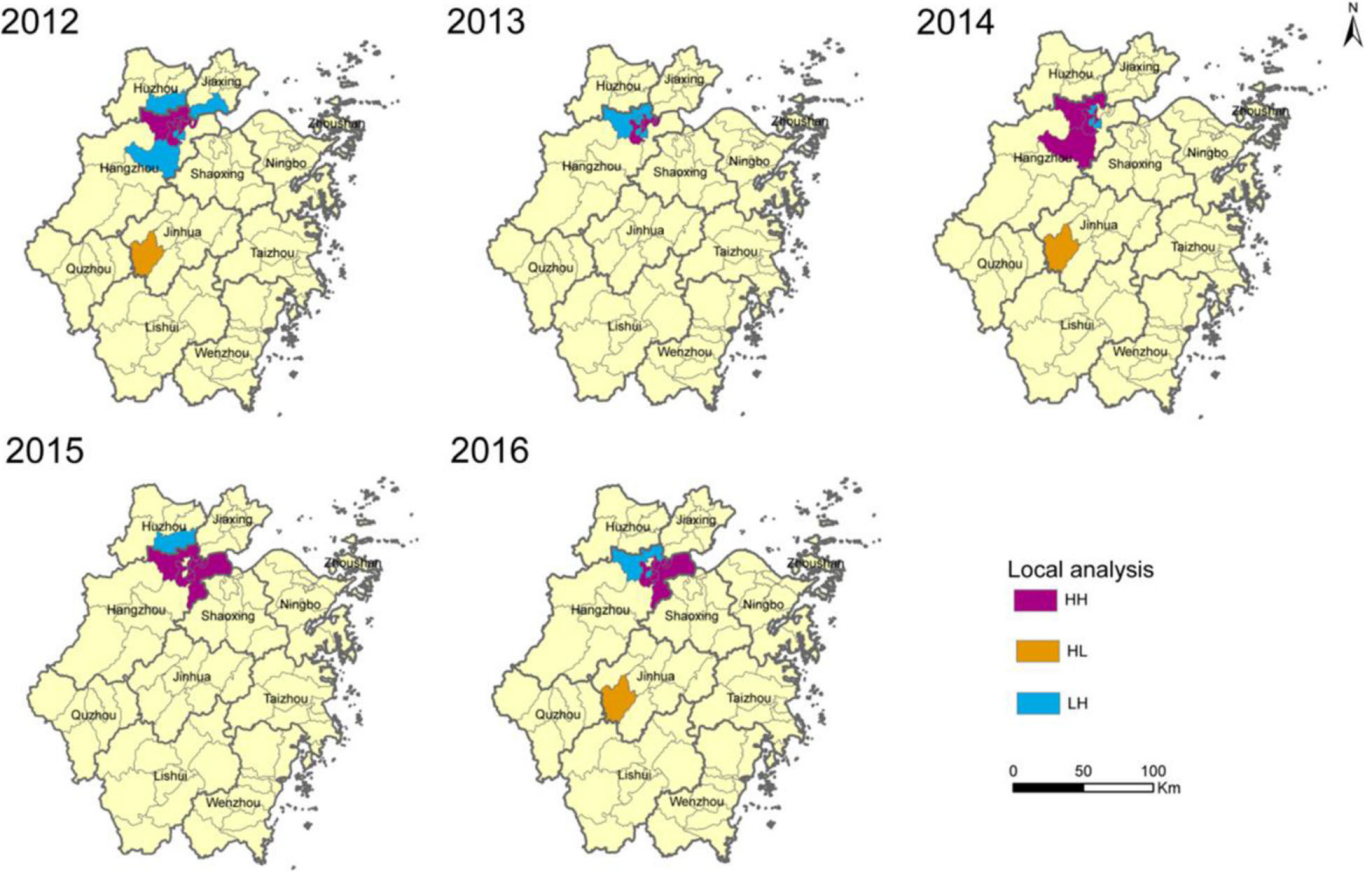
Yearly Local Indicators of Spatial Association cluster maps for HIV-infected students in Zhejiang Province, 2012–2016 (HH: High-High cluster; HL: High-Low outlier; LH: Low-High outlier). The map depicted in the figure is our own

Overall, 72 counties (80.1% of all 89 counties in Zhejiang) reported cases of HIV infection among students between 2011 and 2016, ranging from 25 to 42. The counties of Xiacheng, Shangcheng and Xihu in Hangzhou recorded the most cases during the study period (76, 73, and 65 cases, respectively) (Fig. 1c). Moran’s *I* values ranged from 0.33 to 0.94 (Table 2), indicating that the cases of HIV/AIDS were clustered rather than randomly distributed except for the year of 2011. Furthermore, the local indicators of spatial association analysis identified both HIV/AIDS hotspots (High-High) and outliers in Zhejiang Province (Fig. 2). Hotspots were mainly in the city of Hangzhou but expanded to other areas in later years.

**Table 2:** Spatial autocorrelation of HIV/AIDS positivity among students in Zhejiang Province, 2011–2016

Year	Moran’ *I*	Z score	*p* value
2011	0.11	1.70	*p* > 0.05
2012	0.59	8.92	*p* < 0.01
2013	0.33	5.23	*p* < 0.01
2014	0.39	6.13	*p* < 0.01
2015	0.94	14.09	*p* < 0.01
2016	0.73	10.54	*p* < 0.01

## Discussion

This study provided a comprehensive assessment of the epidemiology of student cases of in Zhejiang Province, China. The proportion of students with HIV/AIDS increased during the period 2011–2016, although HIV disease status and sources of infection of the cases remained stable over time. Homosexual intercourse played a more important role in HIV infection among students compared with the entire HIV-infected population. The spatial distribution of infected students expanded over the study period and showed significant clustering. We also examined the risky sexual behaviors of some newly diagnosed students in detail, and found that most of them had sexual encounters with casual partners.

There may be two reasons for the increase in the proportion of students with HIV/AIDS over the study period. First, homosexual intercourse has been the main mode of transmission among HIV-infected students, as in other provinces in China [16, 22]. Surveys conducted in Liaoning, Beijing and Hefei showed HIV prevalence rates of 1.7–3.0% among MSM students, i.e., up to 50 times higher than that in the general Chinese population (0.057%) [23–26]. Surveys towards the awareness rate of AIDS-related knowledge also showed large difference among regions ranged from 53.38 to 95.7% [6, 11, 27]. MSM students are particularly vulnerable to HIV infection. This study found evidence of sexual networking among students; previously, it was widely thought that HIV-positive social members who transmitted the disease to students. Second, students graduating from high school undergo a psychological transition [28]. Many are curious about the gay community and may experiment sexually with members thereof. A sentinel survey showed that the HIV antibody positivity rate increased from 5.73 to 7.98% in MSM between 2010 and 2015 [29]. However, previous studies in China revealed that condom use in this group was low [30, 31], as also reported in our study, and studies from other countries [32, 33]; this increases the risk of infection via HIV-positive partners. Changes in sexual attitudes and behaviors also contributed to the increase in prevalence [34, 35]. A nationwide cross-sectional survey revealed that 8.3% of 312,016 students were sexually active in the period in 2010–2015, which represented an increase compared to the results obtained before 2010 [4, 28].

Among our sample of students engaged in homosexual intercourse, more than 90% had sex with casual partners. The proportion of HIV-positive students diagnosed via VCT remained steady during the study period. Mobile smartphone applications with global positioning systems enable users to connect with other MSM easily, at any time and in any place, and are thus popular among students seeking new sexual partners. These applications not only include geosocial networking applications targeting MSM (Blued, Jack’d, etc.), but also social media platforms (QQ, WeChat, and Momo) that facilitate risky sexual behavior among students. In a previous survey, over 80% of young MSM indicated that they would be willing to participate in future HIV prevention programs delivered online or via smartphone applications [36]. An outreach and education public health program was conducted in San Mateo County (CA, USA). The number of MSM provided with counseling and education by the program, which was based on the application Grindr, was 14-fold higher than that of a traditional outreach program [37]. Therefore, it is imperative to develop creative digital strategies for HIV prevention among the young MSM.

In this study, detected clusters were distributed within Hangzhou. As Hangzhou is one of the largest and populous city in China, also acted as the capital of Zhejiang Province, it has many universities, at which students obviously congregate. Furthermore, Hangzhou has a relatively high level of economic activity, and MSM may be more sexually active in this city than in other places. A molecular study of MSM recently infected with HIV-l in Zhejiang Province revealed that strains originating from Hangzhou were found throughout the province, suggesting Hangzhou play a central role in the intra-provincial spread of HIV among MSM [38].

The Chinese government published a notification in 2015 to promote the prevention and control of HIV/AIDS in schools, aiming to foster cooperation between the Institutes of Public Health and the Ministry of Education [39]. The importance of timely reporting of epidemiological data on HIV-infected students to the Ministry of Education was emphasized, along with the need to increase the reach of prevention strategies, and improve voluntary consultation testing and behavioral interventions. In the present study, about one third of the students received a diagnosis of HIV through VCT. Nearly half of students had impossible perspective and were not familiar with the precautions against HIV. Increasing the convenience and availability of reaching HIV information targeting testing and prevention for students are important. Online dating applications frequently assessed by students can be greatly promoted such campaigns.

Our results should be interpreted with the following limitations taken into consideration. First, cases of HIV may have been under-reported. Students infected with HIV maybe not diagnosed due to the late for testing. Second, in the *Moran* analyses, we used the number of cases identified each year in each county instead of population-level annual diagnosis rate to detect the clusters. Because the denominator (the total number of students enrolled each year in each county) of the rate was unknown. Third, data collected from the local CDC were not complete; data were only available for some individuals between 2015 and 2016.

There were an estimated 36 million college students in China by the end of 2015 [40]. More targeted and effective interventions are needed to prevent HIV transmission within this vulnerable population.

## Conclusion

This study was performed to assess the epidemiology of HIV among students. The number of cases increased over the study period in Zhejiang Province, with homosexual sexual activity being the main mode of transmission. Sexual encounters with casual partners met through geosocial networking applications targeting MSM and social media messaging platforms were commonly reported. Intensive interventions targeting students are needed; those should promote HIV testing and provide information regarding disease prevention specifically through online dating applications.

## Data Availability

The datasets used and analyzed during the current study are available from the corresponding author (Xiaohong Pan, Email: xhpan@cdc.zj.cn) on reasonable request.

## Abbreviations

HIV: Human immunodeficiency virus
AIDS: Acquired immune deficiency syndrome
MSM: Men who have sex with men
CDC: Center for disease control and prevention
VCT: Voluntary counseling & testing
